# Advances in whole genome sequencing for foodborne pathogens: implications for clinical infectious disease surveillance and public health

**DOI:** 10.3389/fcimb.2025.1593219

**Published:** 2025-04-28

**Authors:** Emílio Gomes, Daniela Araújo, Teresa Nogueira, Ricardo Oliveira, Sónia Silva, Lorena V. N. Oliveira, Nuno F. Azevedo, Carina Almeida, Joana Castro

**Affiliations:** ^1^ INIAV—National Institute for Agrarian and Veterinary Research, Vila do Conde, Portugal; ^2^ LEPABE - Laboratory for Process Engineering, Environment, Biotechnology and Energy, Faculty of Engineering, University of Porto, Porto, Portugal; ^3^ ALiCE - Associate Laboratory in Chemical Engineering, Faculty of Engineering, University of Porto, Porto, Portugal; ^4^ CEB - Centre of Biological Engineering, University of Minho, Braga, Portugal; ^5^ CE3C—Centre for Ecology, Evolution and Environmental Changes & CHANGE, Global Change and Sustainability Institute, Faculty of Sciences, University of Lisbon, Lisboa, Portugal; ^6^ LABBELS – Associate Laboratory, Braga, Guimarães, Portugal; ^7^ Department of Medicine, University of Massachusetts Chan Medical School, Worcester, MA, United States

**Keywords:** outbreak investigation, foodborne pathogens, next-generation sequencing, molecular typing, public health

## Abstract

Foodborne outbreaks affecting millions of people worldwide are a significant and growing global health threat, exacerbated by the emergence of new and increasingly virulent foodborne pathogens. Traditional methods of detecting these outbreaks, including culture-based techniques, serotyping and molecular methods such as real-time PCR, are still widely used. However, these approaches often lack the precision and resolution required to definitively trace the source of an outbreak and distinguish between closely related strains of pathogens. Whole genome sequencing (WGS) has emerged as a revolutionary tool in outbreak investigations, providing high-resolution, comprehensive genetic data that allows accurate species identification and strain differentiation. WGS also facilitates the detection of virulence and antimicrobial resistance (AMR) genes, providing critical insight into the potential pathogenicity, treatment/control options and risks of spreading foodborne pathogens. This capability enhances outbreak surveillance, source tracing and risk assessment, making WGS an increasingly integrated component of public health surveillance systems. Despite its advantages, the widespread implementation of WGS faces several pressing challenges, including high sequencing costs, the need for specialized bioinformatics expertise, limited computational infrastructure in resource-constrained settings, and the standardization of data-sharing frameworks across regulatory and public health agencies. Addressing these barriers is crucial to maximizing the impact of WGS on foodborne disease surveillance. Even so, WGS is emerging as a vital tool in food safety and public health, and its potential to become the gold standard in outbreak detection has been recognized by public health authorities in the USA, the European Union, Australia and China, for example. This review highlights the role of WGS in foodborne outbreak investigations, its implementation challenges, and its impact on public health surveillance.

## 1 Introduction

Foodborne diseases cause approximately 420,000 deaths annually, with children under five accounting for 30% of deaths (Kirk et al., 2015). These diseases are more severe in low- and middle-income countries due to inadequate public health infrastructure (WHO, 2025; Hoffmann et al., 2019). Infections are usually caused by consuming food or water contaminated with bacteria, viruses, fungi, parasites, or toxins produced by them (**Supplementary Table 1**). The growing global food market increases the risk of outbreaks (Grace, 2015), highlighting the need for better detection systems to control foodborne diseases.

Traditional methods, including culture-based, biochemical, immunological and molecular (PCR/qPCR) techniques, are widely used to detect foodborne pathogens (**Supplementary Table 2**) (Priyanka et al., 2016). While effective, they lack the precision required for rapid outbreak management (Quintela et al., 2022). Whole genome sequencing (WGS) addresses these limitations by providing comprehensive genomic data to characterize virulence and antimicrobial resistance traits, distinguish closely related strains, and trace outbreak sources (Li et al., 2021).

In this way, the implementation of WGS has revolutionized the field of foodborne outbreak investigation. The ability to use high-resolution genomic data to complement epidemiological data allows health authorities to respond more quickly and accurately to outbreaks, reducing their spread and decreasing public health problems (Tang and Gardy, 2014). Additionally, WGS has been shown to establish links between distinct outbreaks and/or between geographically distant cases, a feature which traditional methods are too slow or limited to achieve due to the limited genomic information obtained (Koutsoumanis et al., 2019). Despite its benefits, WGS faces challenges such as the need for specialized equipment, bioinformatics expertise and high costs, which limit its widespread adoption (Chrystoja and Diamandis, 2014). This review highlights the critical role of WGS in foodborne outbreak investigations and public health surveillance.

## 2 WGS, a powerful tool in accessing foodborne outbreaks

WGS has emerged as a groundbreaking tool in the field of food safety and public health (Pightling et al., 2018). Its detailed characterization capabilities, including the identification of virulence factors and antimicrobial resistance genes, are expected to lead to its replacement of traditional methods. WGS offers improved surveillance of foodborne pathogens throughout the food supply chain (Allard et al., 2019; Collineau et al., 2019) and enables genetic comparisons to trace pathogen origins (Kovac et al., 2021). **Table 1** highlights the strengths and weaknesses of traditional approaches, emphasizing WGS as a powerful modern tool while recognizing the practicality and accessibility of conventional methods.

**Table 1:** Comparative analysis of strengths and weaknesses of conventional methods and whole genome sequencing (WGS).

Aspect	Conventional Methods	Whole Genome Sequencing (WGS)
**Principle**	Based on phenotypic traits such as culture characteristics, serotyping, biochemical tests, or PCR-based detection (Oluwaseun et al., 2018; Foddai and Grant, 2020).	Sequencing the entire genome to identify pathogens and analyze genetic traits (Allard et al., 2019; Collineau et al., 2019).
**Applications**	Detection, identification, and enumeration of foodborne pathogens (Martinović et al., 2016; Oluwaseun et al., 2018).	Outbreak tracing, source attribution, evolutionary studies, and functional gene analysis (Baert et al., 2021).
**Speed**	Time-consuming (days to weeks, depending on the method) (Gill, 2017; Foddai and Grant, 2020).	Faster results once sequencing infrastructure is established (hours to days) (Scarano et al., 2024).
**Sensitivity and Specificity**	Varies; dependent on culture conditions and the detection method used (Oluwaseun et al., 2018; Foddai and Grant, 2020).	High sensitivity and specificity due to genome-wide analysis (Kovac et al., 2021).
**Data Output**	Qualitative or semi-quantitative results (e.g., presence/absence, counts) (Foddai and Grant, 2020; Saravanan et al., 2020).	Quantitative and comprehensive genetic data (e.g., SNPs, resistome, virulome) (Franz et al., 2016).
**Cost**	Lower initial and operational costs (Gill, 2017; Foddai and Grant, 2020).	High initial cost for sequencing equipment; operational costs depend on scale and throughput; these elevated costs may limit some developing countries, or countries with fewer resources, from accessing this technology (World Health Organization, 2018).
**Advantages**	Cost-effective, well-established, and simple to implement in basic labs (Gill, 2017; Foddai and Grant, 2020).	Provides comprehensive genetic information, including antimicrobial resistance and virulence factors (Allard et al., 2019; Collineau et al., 2019).
**Disadvantages**	Limited accuracy in strain differentiation and inability to detect non-culturable organisms. Relies on viable pathogens; may not detect viable but non-culturable (VBNC) cells or unculturable pathogens (Gill, 2017; Foddai and Grant, 2020).	High initial cost requires advanced infrastructure, expertise, and bioinformatics capabilities; requires high-quality DNA and generates large datasets that need robust bioinformatics pipelines for analysis (World Health Organization, 2018; Brown et al., 2021).

### 2.1 WGS technologies and methodological analysis

The crucial role of WGS is to determine the complete genomic sequence of a given organism. This was only possible with the evolution of second- and third-generation sequencing technologies, which have made this technique very cost and time-effective (Stevens et al., 2022). Second-generation technologies, also known as next-generation sequencing (NGS), sequence thousands of small DNA fragments, which can be assembled to reconstruct the complete genome of the isolates. Recent third-generation sequencing (TGS), usually divided into two main technologies, Pacific Biosciences (PacBio) and Oxford Nanopore Technologies (ONT), uses innovative sequencing principles that allow direct sequencing of long genome sequences and do not require complex post-construction of the genome. TGS also provides rapid sequencing with real-time data analysis, particularly useful in time-sensitive outbreak responses, and allows for direct sequencing of native DNA or RNA, preserving epigenetic modifications (Scarano et al., 2024). Despite these advantages, TGS does have limitations. The raw error rates are generally higher than those of second-generation sequencing platforms such as Illumina, and, while costs for TGS technologies are decreasing, they remain relatively higher for high-throughput applications compared to second-generation sequencing (Ling et al., 2023). The issues associated with sequencing errors can be mitigated by combining second- and third-generation sequencing results to accurately assemble the pathogen genome (Xiao and Zhou, 2020). **Supplementary Table 3** highlights the key differences between Illumina, ONT, and PacBio technologies.

In terms of analysis, the complete genome can then be compared with other known sequences deposited in public health databases, such as those available in PulseNet or The European Surveillance System (TESSy). Additionally, it can be compared with current typing databases, such as core-genome and whole-genome Multilocus Sequence Typing (MLST) (cgMLST/wgMLST), and virulence and/or AMR gene databases for rapid identification of protein-encoding alleles (Franz et al., 2016). This analysis allows the detection of subtle genetic differences that may indicate whether the pathogen comes from a common source or if it is part of a larger outbreak with multiple origins (Rantsiou et al., 2018; Stevens et al., 2022). However, WGS data analysis can be performed using multiple strategies, which introduces variability and reduces the ease of standardization, making it challenging for epidemiologists to interpret the results (Franz et al., 2016). Some of the most used methods include the k-mer approach, which uses the frequency of k-mers to create phylogenetic trees and reference-based methods that align sequenced reads to a common reference genome in order to identify single nucleotide polymorphisms (SNPs) (Franz et al., 2016). In regulatory settings, approaches such as cgMLST are often preferred over SNP-based pipelines because they provide a standardized, reproducible framework based on conserved genomic regions, making data easily comparable across laboratories and jurisdictions, facilitating faster and more reliable outbreak detection, and supporting the integration of genomic data into public health surveillance systems. There are also other alternatives, such as the nucleotide difference approach, which measures differences between reference genomes. The kSNP (k-mer-based Single Nucleotide Polymorphism) method compares the frequency of unique k-mers without prior knowledge but may be affected by mobile genetic elements (Gardner et al., 2015; Franz et al., 2016). Overall, different methodologies for WGS analysis are being developed and shared, leading to limitations in the standardization of the technique (Timme et al., 2019; Uelze et al., 2020).

### 2.2 Global implementation of WGS in foodborne pathogen surveillance: opportunities and challenges

As a new crucial tool for foodborne pathogen surveillance, public health agencies are beginning to implement WGS routinely to track outbreaks more precisely and accurately, enabling more effective responses. In United States of America (USA), the Centers for Disease Control and Prevention (CDC) and the Food and Drug Administration (FDA) created a special program called *GenomeTrakr*, which is responsible for creating a database with the sequences of pathogens from food and environmental samples (Allard et al., 2016). In the United Kingdom (UK), WGS has also been integrated into the national surveillance system and is used by the United Kingdom Health Security Agency (UKHSA) to detect important pathogens, including foodborne pathogens (Gerner-Smidt et al., 2017). Australia has also followed the UK and USA in adopting WGS as a tool to improve foodborne disease surveillance and response by creating a national program of pathogen genomics for public health, the Australian Pathogen Genomics Program (AusPathoGen) (Webb et al., 2024). Recently, the European Union (EU) has adopted a new regulation requiring Member States to conduct WGS on the isolates of five important pathogens (*Salmonella enterica, Listeria monocytogenes, Escherichia coli, Campylobacter jejuni* and *Campylobacter coli*) during the investigations of foodborne illness outbreaks, and sets data-sharing parameters (EU regulation 2025/179) to facilitate foodborne illness outbreak investigations and enable the timely detection of sources and causes. In addition, the European Food Safety Authority (EFSA) has been working with the European Centre for Disease Prevention and Control (ECDC) to develop a joint One Health system that allows the monitoring and control of foodborne diseases across borders, leading to more efficient management and control of the outbreaks (Koutsoumanis et al., 2019). Additionally, in Asia, significant advancements in the use of WGS have been made to enhance food safety and public health. In China, the National Molecular Tracing Network for Foodborne Disease Surveillance (TraNet), launched in 2013, uses WGS for real-time subtyping of foodborne pathogens, which has greatly improved outbreak investigations, source tracking, and cluster analysis across the country (Li et al., 2021). Similarly, in India, WGS has been applied to analyze antimicrobial resistance in milk and dairy-derived pathogens. A study in Anand, Gujarat, assessed the genetic diversity and resistance profiles of these pathogens, demonstrating the potential of WGS for monitoring and managing foodborne diseases in the region (Hati et al., 2024).

Although WGS offers numerous advantages, it is not without its challenges, namely in low- and middle-income countries (LMICs). LMICs are currently facing difficulties in adopting WGS for food safety and AMR surveillance, with one of the main issues being the high costs associated with the required equipment (Apruzzese et al., 2019; Vegyari et al., 2020; Price et al., 2023). In addition, for WGS to work properly, there must be a constant supply of electricity, clean water, and controlled temperatures, some of which are not always reliable in these regions (Vegyari et al., 2020). These added costs make it especially difficult for LMICs to sustain WGS over the long term. These challenges exacerbate the disparity between high-income countries and LMICs in the implementation of WGS for public health (Apruzzese et al., 2019; Vegyari et al., 2020; Price et al., 2023). While wealthier countries are using WGS for food safety and AMR surveillance, LMICs are struggling with resource limitations, widening global inequalities, and hindering compliance with evolving food safety regulations (Apruzzese et al., 2019). Addressing this gap requires urgent investment and innovative solutions such as mobile sequencing labs, the development of regional genomics hubs, specialized training, and the involvement of international aid programs to ensure collaborative and equitable access to the benefits of WGS for all countries (Apruzzese et al., 2019; Vegyari et al., 2020; Price et al., 2023). Another challenge faced in routinely implementing WGS involves having skilled personnel with bioinformatics expertise to analyze and manage the high amount of bioinformatics data generated.

## 3 WGS for foodborne outbreak detection and traceback investigation

**Figure 1:**
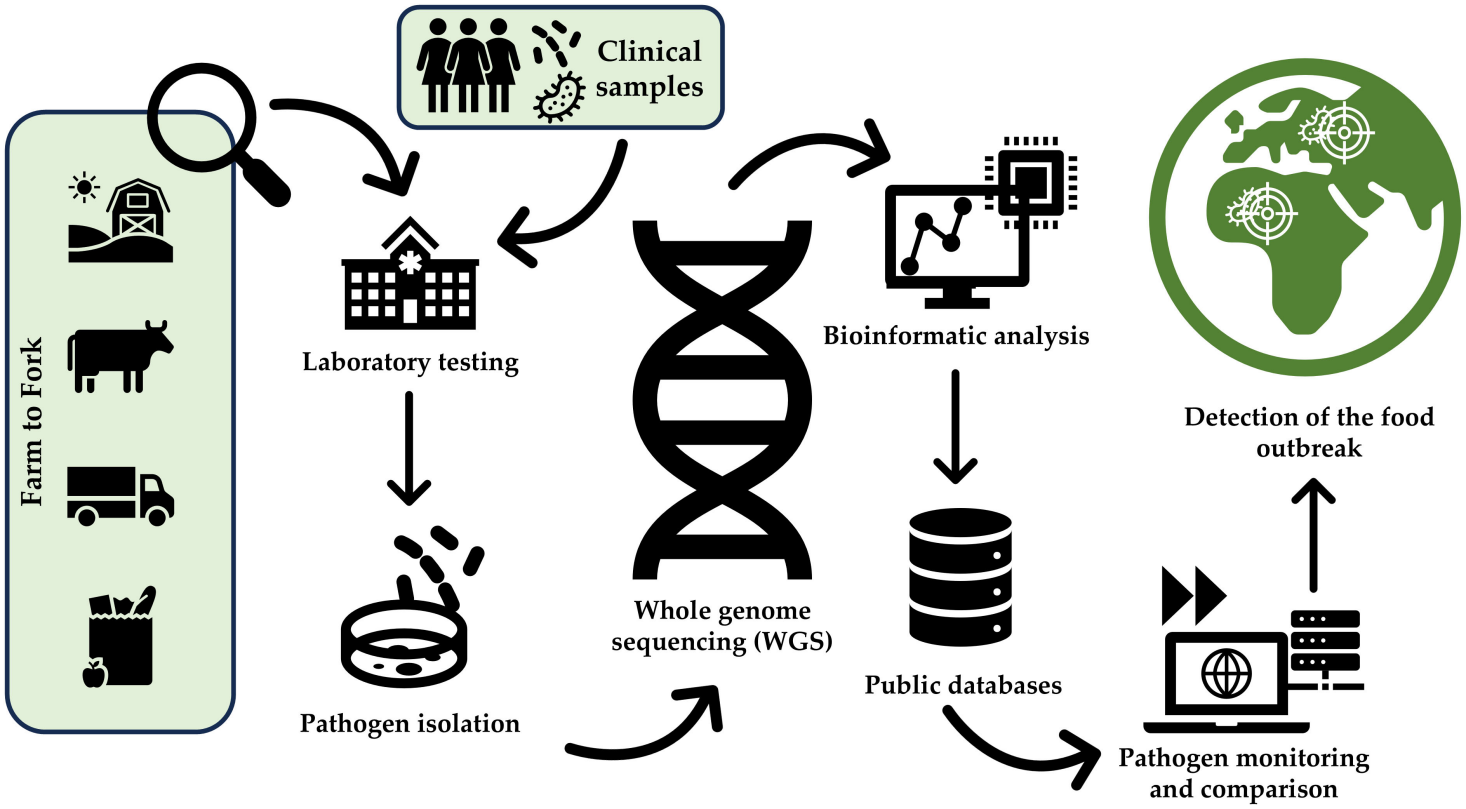
General workflow for foodborne outbreak detection using whole genome sequencing (WGS). The process begins with sample preparation, including the isolation and purification of DNA from food, environmental, or clinical samples. Sequencing is performed using high-throughput platforms to generate raw reads, which undergo quality control and genome assembly. The assembled genomes are annotated to identify genetic features. Integration of genomic and epidemiological data facilitates the identification of outbreak clusters and potential contamination sources, supporting effective public health responses.

The true value of WGS is measured in its real-world application (Morton et al., 2024). It provides a comprehensive analysis of the entire genome, allowing public health authorities to identify outbreak-associated pathogens that are difficult or even impossible to distinguish using traditional typing methods (Chattaway et al., 2019). This process includes the following steps, as also described in **Figure 1**:

- *Sample collection:* Samples can be collected from patients, food, and environmental sources (Chen et al., 2020). The microorganisms are then isolated using culturomics, and the DNA/RNA is extracted and purified.
- *Sequencing and analysis*: The DNA/RNA of isolated pathogens is sequenced, and the raw data is processed and analyzed to identify/characterize the species and even determine if there are any similarities between different samples (Chen et al., 2020).
- *Databases comparison*: The outbreak strains are compared and deposited in national and international databases by the authorities — this is followed by statistical analysis to identify the patterns that can further be used to trace back to the suspected source of contamination (Chen et al., 2020).

This method of matching the outbreak strains more quickly, and perhaps even from across borders, allows the health authorities to take early mitigation action before the outbreak spreads further (Holmes et al., 2015).

In addition to the consequences for human health, an outbreak can have a significant impact on world trade (Tibebu et al., 2024). In some cases, the affected countries/regions may be subject to trade bans with several countries or even more stringent border controls (Jansen et al., 2019). This can have a detrimental effect on food logistics and, by extension, the global/national economy. The various consequences of an outbreak highlight the need for the rapid recognition of such events and the subsequent rapid implementation of appropriate containment measures. By enabling timely interventions, WGS significantly reduces the financial burden associated with foodborne outbreaks. Early detection and traceback minimize healthcare costs, mitigate losses from product recalls, and prevent trade disruptions. This proactive approach not only enhances public health protection but also strengthens the economic resilience of the food industry by reducing the overall impact of outbreaks.

Thus, WGS has been essential in tracing outbreak sources, controlling foodborne disease spread, and analyzing pathogen virulence. **Supplementary Table 4** highlights key studies demonstrating its application in outbreak detection and virulence analysis. For instance, in 2022, there was a *Salmonella* Typhimurium outbreak in the USA, where WGS was crucial for the genetic characterization of *Salmonella* isolates, tracing the possible source of the outbreak, and linking it to a cantaloupe farm, which enabled public authorities to take the necessary actions (Seelman Federman et al., 2024). Additionally, WGS also made it possible to determine antimicrobial resistance, detect virulence factors, and perform phylogenetic analysis, providing insights into the epidemiology and genetic characteristics of *Salmonella* isolates from poultry meat in Pakistan (Siddique et al., 2024).

## 4 Use of WGS in the routine inspection process

WGS methodologies have the potential to become an essential tool in food surveillance. By determining the genetic information of harmful pathogens, health authorities can not only detect their presence, allowing early identification of potential outbreaks, but also identify genes that encode AMR and/or virulence factors (Kovac et al., 2021). The use of sequence alignment algorithms through reliable open-access databases of genes and mutations, such as ResFinder or VirulenceFinder (hosted by the Center for Genomic Epidemiology at the Technical University of Denmark), makes it possible to identify and predict antimicrobial resistance and virulence phenotypes in a simple and effective way (Stevens et al., 2022).

WGS fulfills all the requirements to become a standard procedure in food safety inspection practices, which is already practiced by some public health and regulatory agencies, such as the CDC/FDA and the ECDC/EFSA (Brown et al., 2019). During these routine inspections, food samples from various stages of production, from raw ingredients, in-process samples, and final products, can be tested for pathogens using WGS. Moreover, environmental swabs from food production facilities and processing equipment can also be analyzed (Lakicevic et al., 2023). The data obtained from WGS will allow the identification and comparison of species and strains of the pathogens, enabling the detection and comparison of minimal differences between samples and/or related bacteria (Stevens et al., 2022).

Future advances in sequencing technologies, such as the Oxford Nanopore’s MinION, which is a portable sequencing device designed for used in the field, will enable real-time, on-site WGS for public health inspection (Billington et al., 2022; Samdarshi, 2024). Additionally, AI-driven data analysis will simplify WGS interpretation and improve outbreak detection with faster and more accurate source identification. As these technologies evolve, costs will decrease, encouraging wider adoption in global food safety surveillance (Du and Guo, 2022; Qian et al., 2023).

## 5 WGS data sharing

### 5.1 Standardization of workflows to ensure the generation of comparable results

The introduction of WGS has revolutionized cross-border surveillance and outbreak investigation, establishing bioinformatic analysis of pathogen genomes as a potential gold standard. However, effective collaboration between microbiologists and informaticists and standardized methodologies are crucial for generating comparable data across laboratories (Gilchrist et al., 2015; Koutsoumanis et al., 2019).

At the European level, various initiatives have been launched to standardize life sciences data management across the EU. For example, the EFSA has provided technical guidance for implementing the WGS One Health analytical pipeline (Costa et al., 2022). However, harmonizing data across the different sectors involved in this initiative (e. g. food safety, veterinary, and public health) has presented challenges. A key issue has been ensuring data compatibility between distinct pipelines, particularly regarding the integration of pathogen sequencing data from various sources. To address this, EFSA has worked closely with the European Centre for Disease Prevention and Control (ECDC) to align analytical methods and metadata standards, although inconsistencies in data reporting remain a challenge (ECDC et al., 2019).

The One Health WGS system connects two analytical pipelines: the food and veterinary pipeline, which provides data to EFSA’s One Health WGS analytical pipeline to generate derived data for studying foodborne outbreaks; and the public health pipeline, managed by ECDC, which receives and uses public health data to its analyses (ECDC et al., 2019). While this system aims to centralize pathogen surveillance, one of the challenges has been achieving widespread participation from laboratories across different countries, each with varying capacities in terms of technical infrastructure and expertise.

In overcome these challenges, ELIXIR, the European life sciences infrastructure, has initiated the ELIXIR-CONVERGE project, funded by the European Commission, to harmonize life science data management. This project offers a toolkit to make research data publicly accessible and expands scientists’ access to diverse datasets, including food-related information (Durinx et al., 2017).

Furthermore, the European Commission also established the Inter-European Union Reference Laboratories (EURLs) Working Group on NGS. This group aims to promote NGS adoption within EURL networks, enhance NGS capacity across the EU, and facilitate collaboration among the EURLs, EFSA, and ECDC. It includes all EURLs focusing on microbiological contamination in food and feed (Michelacci et al., 2023).

Additionally, the USA has made notable progress in using NGS for pathogen surveillance, but its approach is less centralized compared to the European Union’s efforts. A key initiative in the USA is the CDC’s PulseNet, a network that facilitates the sharing of WGS data between federal and state laboratories to track foodborne outbreaks (Tolar et al., 2019). Although PulseNet significantly improves outbreak detection, it operates independently of other initiatives like the FDA’s GenomeTrakr, which is more focused on genomic surveillance of foodborne pathogens (Allard et al., 2016; Kubota et al., 2019).

Overall, while both the EU and the USA have made significant progress in integrating WGS into public health surveillance, the EU adopts a more centralized approach through EFSA, ECDC, and ELIXIR, promoting uniform data management and cross-border collaboration. The EU’s more centralized approach helps mitigate some of the standardization issues but faces challenges due to differing national capacities and regulations. In contrast, the USA’s decentralized model, exemplified by PulseNet and GenomeTrakr, allows for more flexibility but may hinder cross-state and cross-sector integration of data. As both regions move forward, overcoming these challenges will be crucial for maximizing the global impact of WGS in pathogen surveillance and food safety efforts (Allard et al., 2016; Durinx et al., 2017; Tolar et al., 2019).

### 5.2 Interoperable data for foodborne outbreak surveillance

Genomic sequences should follow the Findable, Accessible, Interoperable, and Reusable (FAIR) Principles, ensuring standardized, interoperable data sharing across global platforms to enhance foodborne outbreak tracking and pathogen surveillance (Hovig et al., 2021). Researchers have two primary options for managing the data they generate:

i) Direct Submission, in which they can process, store, and submit their data directly to international repositories or databases. Research results, including data and metadata for outbreak tracking, should be shared in FAIR format. Platforms like GitHub are widely used to share bioinformatics pipelines, analysis scripts and metadata, while processed genomic data can subsequently be deposited and shared in established genomic repositories such as the European Nucleotide Archive (ENA) and the National Center for Biotechnology Information (NCBI). These platforms are crucial for direct data submission, providing standardized formats that facilitate data integration into ongoing public health initiatives and global pathogen surveillance systems.
ii) Data Brokerage Model in which an intermediary can curate the raw data, analyze it according to standardized guidelines, store it, and share processed, de-identified sequence and metadata needed for outbreak tracing with public health databases and international repositories (Singh et al., 2024).

EU Member States are required to provide the ECDC and/or EFSA with scientific and technical data relevant to its mission promptly. The ECDC manages the TESSy, a platform for collecting, analyzing, and disseminating surveillance data on infectious diseases across Europe (Kramarz et al., 2014; Walle et al., 2019). Competent national authorities supply comparable and compatible data on the epidemiological surveillance of communicable diseases and related health issues to this network. EFSA is also planning to create a platform dedicated to WGS data, which could be shared with ECDC to create a single database encompassing food and human outbreak data.

The implementation of these databases, such as ECDC/TESSy platform, facilitates sequence data sharing, integrative analysis, and reporting. Data providers upload sequences, metadata, and epidemiological data to a secure storage solution with controlled, reliable, long-term access. Genomic and epidemiological data are presented together with derived data, such as allele identification, genetic markers, strain nomenclature, and genetic distances generated and visualized for comprehensive analysis (Hawkins et al., 2010; Walle et al., 2019).

## 6 Conclusions

WGS has revolutionized foodborne outbreak detection with high-resolution genetic data for pathogen identification, traceability and AMR/virulence profiling. Despite challenges such as cost and infrastructure, the benefits outweigh the limitations, solidifying WGS as a crucial approach in foodborne pathogen investigation. Advances in technology and data sharing will enhance accessibility and strengthen food safety and public health efforts. Looking to the future, the integration of artificial intelligence for advanced data analysis and the development of portable sequencing devices hold significant potential to expand the reach and improve the effectiveness of WGS. These innovations are poised to further revolutionize global foodborne pathogen surveillance and offer exciting opportunities for the continued development and increased accessibility of WGS technologies in diverse settings.
