# Supplementary material for: Advances in whole genome sequencing for foodborne pathogens: implications for clinical infectious disease surveillance and public health
Source: Front Cell Infect Microbiol. 2025 Apr 28;15:1593219. doi: 10.3389/fcimb.2025.1593219 (PMC12066639; doi:10.3389/fcimb.2025.1593219)
Supplement: Supplementary file 1 [file Table1.docx]

Supplementary Material

Supplementary Table 1. Some of the most common foodborne pathogens, the typical food sources, the associated disease and incidence

| **Pathogen** | **Foodborne pathogen species/type** | **Source** | **Disease** | | **Incidence**  **(2021 and 2022)** | **Burden (annually)** | **Reference** |
| --- | --- | --- | --- | --- | --- | --- | --- |
| ***Alternaria* spp.** | *Alternaria alternata* | Cereals, fruits, vegetables | Allergic reactions, gastrointestinal diseases, carcinogenic potential | | NA | ~ several $ billion | (Saleh et al., 2024) |
| ***Arcobacter* spp*.*** | *A. butzleri* | Pre-cut ready-to-eat vegetables | Acute diarrhea, abdominal pain, nausea, vomiting | | NA | NA | (Mottola et al., 2016; Ruiz de Alegría Puig et al., 2023) |
| ***Aspergillus* spp**. | *Aspergillus flavus*  *Aspergillus parasiticus*  *Aspergillus nomius* | Peanuts, spices. nuts | Aflatoxicosis | | 11,6 cases per 100 000 people in the EU | $ 11,6 bilion | (Pickova et al., 2021) |
| ***Bacillus* spp.** | *B. cereus* | Meat products, soups, vegetables, puddings, sauces, milk and milk products | Emetic and diarrheal syndrome | | 0.07 cases per 100 000 people in the EU | ~several million € | (Bintsis, 2017; Food Safety Authority and Centre for Disease Prevention, 2024) |
| ***Campylobacter* spp.** | *C. jejuni C. coli* | Poultry, unpasteurized milk | Campylobacteriosis | | 43.1 cases per 100 000 people in the EU | ~ 2.4 billion € | (Heredia and García, 2018; Food Safety Authority and Centre for Disease Prevention, 2024) |
| ***Clostridium* spp.** | *C. perfringens* | Beef and chicken (improperly stored/ undercooked) | | Gastroenteritis | 0.01 cases per 100000 people in the EU | ~tens of million € | (Kirk et al., 2015; Bintsis, 2017; Food Safety Authority and Centre for Disease Prevention, 2024) |
|  | *C. difficile* | Seafood,  vegetables, meat | Antibiotic-associated diarrhea | | NA | ~ 3.5 billion € | (Rodriguez et al., 2016; Food Safety Authority and Centre for Disease Prevention, 2024) |
| ***Cronobacter* spp.** | *C. sakazakii* | Dried milk powder, dried meats, vegetables, nuts, dried flours and spices | Acute  gastroenteritis | | NA | NA | (Bintsis, 2017; Yong et al., 2018) |
| ***Cyanobacteria*** | Microcystins produced by *Anabaena, Aphanocapsa, Hapalosphon, Microcystis, Nostoc, Oscillatoria, Planktothrix*;  Saxitoxins produced by *Anabaena, Aphanizomenon, Cylindrospermopsis, Lyngbya* | Bioaccumulation of  cyanotoxins in seafood, aquatic  products, food animals, and crops | Gastrointestinal pain, liver inflammation, pneumonia, and dermatitis | | NA | $50 million of HABs (harmful algae biomes) | (Socioeconomic – Harmful Algal Blooms, n.d.; Hardy et al., 2015; Lee et al., 2017) |
| ***E. coli*** | Enterotoxigenic *E. coli* (ETEC) | Raw vegetables | Traveler’s  diarrhea | | NA | ~several million € | (Kirk et al., 2015; Yang et al., 2017; Food Safety Authority and Centre for Disease Prevention, 2024) |
|  | Shiga toxin-producing *E. coli* (STEC) Serogroup O157 | Raw products such as meat, milk and vegetables | Severe hemorrhagic colitis | | 2.1 cases per 100000 people in the EU | ~1.3 billion € | (Majowicz et al., 2014; Heredia and García, 2018; Food Safety Authority and Centre for Disease Prevention, 2024) |
| ***Klebsiella*** | *K. pneumoniae* | Raw meat and vegetables, ready to eat food | Bacteremia, pneumonia, and urinary tract infection | | NA | ~2 billion € | (Hartantyo et al., 2020; Riley, 2020; Food Safety Authority and Centre for Disease Prevention, 2024) |
| ***Listeria* spp.** | *L. monocytogenes* | Milk products, meat products, and fresh produce, seafood | Listeriosis | | 0.62 cases per 100 000 people in the EU | ~4.5 billion € | (Ravindhiran et al., 2023; Food Safety Authority and Centre for Disease Prevention, 2024) |
| ***Norovirus*** |  | Mollusks | Gastroenteritis | | 0.07 cases per 100000 people in the EU | ~1.8 billion € | (Food Safety Authority and Centre for Disease Prevention, 2024) |
| ***Salmonella* spp.** | *S.* Typhimurium  *S.* Enteritidis | Poultry derived products, dairy products | Localized gastroenteritis | | 15.3 cases per 100000 people in the EU | ~3 billion € | (Jajere, 2019; Food Safety Authority and Centre for Disease Prevention, 2024; Lamichhane et al., 2024) |
| ***Shigella* spp*.*** | *S. sonnei* | Contaminated salad vegetables and fresh herbs | Shigellosis | | <0.01 cases per 100000 people in the EU | ~ tens of million € | (Jenkins et al., 2023; Food Safety Authority and Centre for Disease Prevention, 2024; Yao et al., 2024) |
| ***Staphylococcus* spp.** | *S. aureus* | Milk products, meat products,  salads, bakery products | Vomiting,  abdominal pain, and stomach cramps | | 0.03 cases per 100000 people in the EU | ~several million € | (Fetsch et al., 2014; Li et al., 2022; Food Safety Authority and Centre for Disease Prevention, 2024) |
| ***Toxoplasma*** | *T. gondii* | Raw meat and milk, fresh  produce, shellfish | Toxoplasmosis | | 5.6 cases per 100000 people in the EU | ~250-500 million € | (Food Safety Authority and Centre for Disease Prevention, 2024) |
| ***Vibrio* spp.** | *V. parahaemolyticus* | Seafood | Acute gastroenteritis | | <0.01 cases per 100000 people in the EU | NA | (Zhong et al., 2022; Food Safety Authority and Centre for Disease Prevention, 2024) |
| ***Yersinia* spp.** | *Y. enterocolitica* | Raw milk and pork products | Yersiniosis | | 2.2 cases per 100000 people in the EU | ~several million € | (Bintsis, 2017; Food Safety Authority and Centre for Disease Prevention, 2024) |

*Abbreviations:* NA: Not available

# Supplementary Table 2. Conventional typing methods used for the detection of foodborne pathogens

| **Method** | **Advantages** | **Limitations** | **Examples** |
| --- | --- | --- | --- |
| **Culture-Based Methods** | -Well-established and easy to use;  - Less expensive;  - Quantification by counting the number of colonies;  - Allow the isolation of microorganisms for further analysis. | -Lower sensitivity for some pathogens;  -Time-consuming and laborious cultures are disadvantageous;  -Not suitable for rapid detection;  - Not all pathogens grow easily;  - Trained staff requirement. | Culture of some of the most common gastrointestinal bacterial pathogens is a typical measure in outbreak detection (Motladiile et al., 2019). For instance, this method is used to distinguish *E. coli* O157:H7 on Sorbitol MacConkey agar from other strains and Cefsulodin-Irgasan-Novobiocin agar is used to differentiate *Yersinia enterocolitica* from other bacteria (Priyanka et al., 2016). |
| **Biochemical tests** | - Relatively quick, which can expedite preliminary pathogen identification;  - Detect specific metabolic characteristics unique to the pathogen;  - Inexpensive and easy to use. | - Limited sensitivity;  - Variability of results (similar metabolic reactions can lead to false negatives/positives results);  - Labor-intensive when multiple biochemical tests are required. | The IMViC tests (Indole, Methyl Red, Voges-Proskauer, and Citrate) are used as a complement to the culture-based methods and allow to detect and distinguish *Salmonella* from other *Enterobacteriaceae* (Nazari Moghadam et al., 2023).  These tests are also useful to detect *Enterobacteriaceae*, such as *Shigella* in food products such as dairy and meat, and their presence may indicate unsanitary processes (Saravanan et al., 2020; Nazari Moghadam et al., 2023). |
| **Immunological assays** | - High specificity;  - Easy to perform;  - Less labor-intensive and rapid results;  - Large number of samples that can be processed at once. | - Limited sensitivity;  - Possible cross-reactivity with non-target microorganisms;  - More expensive than culture-based methods;  - Unable to differentiate viable from non-viable cells. | ELISA is used to detect specific antibodies to help confirm foodborne pathogens infections, such as Toxoplasmosis (Ekman et al., 2012). This method is also a rapid and easy way to detect *Salmonella enterica* in food samples, reducing the time required for an outbreak detection (Di Febo et al., 2019). |
| **Molecular methods (PCR/qPCR)** | - High sensitivity, allowing the detection of low levels of pathogens;  - High specificity;  - It is possible to identify multiple microbial groups;  - Rapid results, often within a few hours. | - High cost and technical expertise required;  - Primer design must be well-concept  - Cannot discriminate between viable and non-viable cells;  - Sensitive to contamination and inhibitors (false positives and false negatives). | PCR is used to rapidly and simultaneously detect several key foodborne pathogens in samples, such as *E. coli,* *L. monocytogenes, S. aureus* and *S. enterica* (Boukharouba et al., 2022).  This technique is also used for the detection of foodborne viruses in samples, such as *Norovirus*, and it is useful for the quick detection of *Salmonella* with the amplification of the 23S rRNA genes (Souii et al., 2016). |

**Supplementary Table 3.** Comparison of key Next-Generation Sequencing (NGS) and third-generation sequencing platforms for foodborne pathogen detection and genome analysis

| Platform | Read Length | Accuracy | Cost per Gb | Typical Use Cases | References |
| --- | --- | --- | --- | --- | --- |
| Illumina | Short (75–300 bp) | >99.9% (HiFi) | Low | High-throughput WGS, metagenomics, outbreak surveillance | (Goodwin et al., 2016; van Dijk et al., 2018) |
| ONT (Nanopore) | Long (up to >1 Mb) | 90–98% (raw), >99.9% (with polishing) | Moderate | Real-time sequencing, rapid pathogen detection, portable applications | (Logsdon et al., 2020; Wang et al., 2021) |
| PacBio (High-Fidelity, HiFi sequencing) | Long (10–25 kb) | >99.9% (HiFi) | High | Complete genome assembly, complex genomic regions, AMR gene detection | (Rhoads and Au, 2015; Wenger et al., 2019) |

**Supplementary Table 4.** Foodborne outbreaks from the last decade and the application of WGS

| Pathogen | Source | Country (ies) | **WGS application** | Sequencing/Assembly methodology | Year | Reference |  |
| --- | --- | --- | --- | --- | --- | --- | --- |
| WGS for traceback investigation | | | | | | | |
| *Bacillus cereus* | | Refried beans | USA | Characterization of the pathogen, identification of genetic relationships and improvement of outbreak detection and investigation. | Paired-end seq. Illumina MiSeq/ de novo assembly | 2016 | (Carroll et al., 2019) |
| *Brucella melitensis* | | Camel Milk | Israel | Identification of the outbreak and tracing of the possible origin. |  |  | (Bardenstein et al., 2021) |
| *Campylobacter jejuni* | | Chicken liver pâté | Sweden |  | Paired-end seq. Illumina MiSeq/ de novo assembly | 2012 | (Lahti et al., 2017) |
|  |  |  | Australia |  | Paired-end seq. Illumina NextSeq 500/ reference- | 2013 | (Moffatt et al., 2016) |
|  |  | Raw milk | Japan |  |  | 2018 | (Ohno et al., 2023) |
| *Clostridium botulinum* | | Commercial Nacho Cheese Sauce | USA |  | Paired-end seq. Illumina MiSeq/ de novo assembly | 2017 | (Rosen et al., 2020) |
|  |  | Vacuum-Packed Ambient-Stored Chili Chicken Feet | China |  |  | 2019 | (Dong et al., 2022) |
| *E. coli -STEC* | | Meat | USA | Identification of high genetic diversity in the pathogen from retail meat and tracing the possible outbreak source. | Not specified | 2019 | (2019 E. coli Outbreak Linked to Ground Beef, \| CDC, 2019) |
| *Listeria*  *monocytogenes* | | Crabmeat | UK | Identification of the outbreak and tracing of the possible origin. | Not specified/ reference-based assembly | 2013 | (Elson et al., 2019) |
|  |  | Smoked fish | Denmark |  | Paired-end seq. Illumina MiSeq/ reference-based assembly | 2013-2015 | (Gillesberg Lassen et al., 2016) |
|  |  | Frozen vegetables | USA |  | Not specified/ reference-based assembly | 2016 | (Madad et al., 2023) |
|  |  | Unpasteurized milk | USA |  | Not specified | 2016 | (Nichols et al., 2020) |
| *Salmonella*  Bareilly | | Dried eggs | Czech Republic, Slovakia | Identification of the outbreak and tracing of the possible origin. | Not Specified/ de novo assembly | 2017 | (Labská et al., 2021) |
| *Salmonella* Bovismorbificans | | Uncooked ham products | The Netherlands |  | Paired-end seq. Illumina MiSeq/ de novo assembly |  | (Brandwagt et al., 2018) |
| *Salmonella* Braenderup | | Melons | UK |  | Paired-end seq. Illumina HiSeq 2500/ reference-based assembly | 2021 | (Chan et al., 2023) |
| *Salmonella* Derby | | Pork meat | Germany |  | Paired-end seq. Illumina MiSeq/ reference-based assembly | 2013-2014 | (Simon et al., 2018) |
| ***Salmonella***  **Enteritidis** | | Eggs | UK and Spain |  | Paired-end seq. Illumina HiSeq 2500/ reference-based assembly | 2015 | (Inns et al., 2017) |
|  |  |  | South Africa |  | Not specified | 2018 | (Smith et al., 2020) |
| *Salmonella*  Heidelberg | | Chicken | USA | Enhanced outbreak investigation through phylogenetic analysis, linking human and food isolates to common sources and supporting traceback investigations. | Paired-end seq. Illumina MiSeq/ reference-based assembly | 2014 | (Crowe et al., 2017) |
| *Salmonella* Mbandaka | | Not clear | Australia | Differentiated outbreak cases and assisted authorities in investigating potential sources. | Paired-end seq. Illumina NextSeq 500/ reference-based assembly | 2016 | (Lindsay et al., 2018) |
| *Salmonella*  Montevideo | | Restaurant, unclear food source | Canada | Identification of the outbreak and tracing of the possible origin. | Not specified/ de novo assembly | 2020-2021 | (Paradis et al., 2023) |
| *Salmonella*  Newport | | Frozen cooked crayfish | Sweden |  | Paired-end seq. Illumina MiSeq/ hybrid assembly | 2019 | (Mörk et al., 2022) |
| *Salmonella*  Oranienburg | | Onions | Mexico |  | Not specified/ de novo assembly | 2021 | (Mitchell et al., 2024) |
| *Salmonella* Typhimurium | | Leafy greens | USA | Identification of the outbreak and tracing of the possible origin. | Not specified/ reference-based assembly | 2021 | (McClure et al., 2023) |
|  |  | Cantaloupes |  |  |  | 2022 | (Federman et al., 2024) |
| *Salmonella* Weltevreden | | Frozen Precooked Shrimp |  |  | Not specified/ de novo assembly | 2021 | (Jenkins et al., 2024) |
| *Salmonella*  Poona | | Rice-based infant formula | France | Identification of the outbreak, tracing of the possible origin and link to previous outbreaks on the region. | Paired-end seq. Illumina NextSeq 500/ de novo assembly | 2018-2019 | (Jones et al., 2019) |
| *Shigella sonnei* | | Spring Onions | UK | Identification of the outbreak and tracing of the possible origin. | Paired-end seq. Illumina NextSeq 1000/ reference-based assembly | 2021 | (Jenkins et al., 2023) |
| *Streptococcus equi* subsp.  *zooepidemicus* | | Unpasteurized Dairy | Italy | Identification of the outbreak and tracing of the possible origin. | Paired-end Illumina and Oxford Nanopore MinION/ hybrid assembly | 2021-2022 | (Bosica et al., 2023) |
| *Vibrio parahaemolyticus* | | Seafood | China | Identification of the outbreak and tracing of the possible origin. | Not specified/ reference-based assembly | 2021 | (Zhang et al., 2022) |
| *Yersinia enterocolitica* | | Pasteurized Milk | USA |  |  | 2019 | (Gruber et al., 2021) |
|  |  | Fresh spinach | Sweden and Denmark |  |  |  | (Espenhain et al., 2019) |
| WGS for virulence studies | | | | | | | |
| *A. butzleri* | | Milk, meat, and ready-to-eat vegetables | Lithuania | Identification of genetic diversity, AMR mechanisms, and virulence profiles of this emerging pathogen. | Paired-end seq. Illumina MiSeq/ de novo assembly | 2022 | (Uljanovas et al., 2023) |
| *Clostridium perfringens* | | Poultry, vegetables | France | Phylogenomic reconstruction of the pathogen, identification of strain diversity and epidemiological link between outbreaks. | Paired-end seq. Illumina NextSeq 500/ de novo assembly | 2013-2017 | (Abdelrahim et al., 2019) |
| *C. sakazakii* | | Infant Formula | China | Identification, characterization and tracing of the pathogen contamination in production facilities and products. | Paired-end seq. Illumina HiSeq 2000/ de novo assembly | 2012-2018 | (Tong et al., 2024) |
| *Cyanobacteria* | | NA | NA | Discovery of genes linked to bioactive compound synthesis, multidrug resistance, heavy metal resistance, and virulence factors. |  | 2018-2019 | (Nitnaware et al., 2021) |
| *E. coli -ETEC* | | Not determined | Korea | Analysis of genomic characteristics, identification of virulence factors, and determination of phylogenetic relationships between ETEC O159 strains from patients. | Paired-end seq. Illumina HiSeq 2000/ de novo assembly | 2003-2011 | (Chung et al., 2019) |
|  |  | Meat | China | Identification of high genetic diversity in the pathogen from retail meat and tracing the possible outbreak source. | Paired-end seq. Illumina HiSeq 4000/ de novo assembly | 2021-2023 | (Wang et al., 2024) |
| *Klebsiella*  *pneumoniae* | | Raw Milk | China | Identification of the pathogen and its AMR genes. | Paired-end seq. Illumina HiSeq 2500 and Oxford Nanopore Technologies MiniON/ hybrid assembly | 2021 | (Ye et al., 2024) |
| *Mucor circinelloides* | | Yogurt | USA | Generation of reference genomes and comparison of genetic differences between *Mucor circinelloides* isolates | Paired-end seq. Illumina HiSeq 2000/ reference-based assembly | 2013 | (Lee et al., 2014) |
| *Norovirus* | | Raw foods, such as vegetables, fruits and mollusks | USA | Identification of the pathogen and differentiation of strains from different outbreaks within the same genotype. | Paired-end seq. Illumina MiSeq/ Not Specified | 2012-2019 | (Silva et al., 2021) |
| *Salmonella*  Reading | | Chicken meat | Canada, Pakistan | Characterization of genetic features, identification of AMR genes and plasmids, and phylogenetic analysis of ciprofloxacin-resistant strains from poultry. | Paired-end seq. Illumina MiSeq/ de novo assembly | 2014-2015 | (Siddique et al., 2024) |
| *Staphylococcus aureus* | | “Sushi”; fish | Japan | Identification and characterization of the virulence factors AMR genes, and genomic structure of the pathogen. | Paired-end Illumina and Oxford Nanopore MinION/ hybrid assembly | 2016 | (Umeda et al., 2021) |
| *Toxoplasma gondii* | | Leafy vegetables or fruit, seafood, undercooked meat | USA | Direct detection and characterization of the pathogen from environmental and food samples, enhancing genomic epidemiology and surveillance for food and waterborne diseases. | Paired-end Illumina NextSeq/ reference-based assembly | 2022-2023 | (Sundararaman et al., 2024) |

**References**

2019 *E. coli* Outbreak Linked to Ground Beef | *E. coli* Infections | April 2019 | *E. coli* | CDC (n.d.). Available at: https://archive.cdc.gov/www_cdc_gov/ecoli/2019/o103-04-19/index.html (Accessed November 15, 2024).

Abdelrahim, A. M., Radomski, N., Delannoy, S., Djellal, S., Le Négrate, M., Hadjab, K., et al. (2019). Large-Scale genomic analyses and toxinotyping of *Clostridium perfringens* implicated in foodborne outbreaks in France. *Front Microbiol* 10, 777. doi: 10.3389/FMICB.2019.00777/FULL

Bardenstein, S., Gibbs, R. E., Yagel, Y., Motro, Y., and Moran-Gilad, J. (2021). Brucellosis Outbreak Traced to Commercially Sold Camel Milk through Whole-Genome Sequencing, Israel. *Emerg Infect Dis* 27, 1728. doi: 10.3201/EID2706.204902

Bintsis, T. (2017). Foodborne pathogens. *AIMS Microbiol* 3, 529. doi: 10.3934/MICROBIOL.2017.3.529

Bosica, S., Chiaverini, A., De Angelis, M. E., Petrini, A., Averaimo, D., Martino, M., et al. (2023). Severe *Streptococcus equi* Subspecies *zooepidemicus* Outbreak from Unpasteurized Dairy Product Consumption, Italy. *Emerg Infect Dis* 29, 1020. doi: 10.3201/EID2905.221338

Boukharouba, A., González, A., García-Ferrús, M., Ferrús, M. A., and Botella, S. (2022). Simultaneous Detection of Four Main Foodborne Pathogens in Ready-to-Eat Food by Using a Simple and Rapid Multiplex PCR (mPCR) Assay. *International Journal of Environmental Research and Public Health 2022, Vol. 19, Page 1031* 19, 1031. doi: 10.3390/IJERPH19031031

Brandwagt, D., van den Wijngaard, C., Tulen, A. D., Mulder, A. C., Hofhuis, A., Jacobs, R., et al. (2018). Outbreak of *Salmonella* Bovismorbificans associated with the consumption of uncooked ham products, the Netherlands, 2016 to 2017. *Euro Surveill* 23. doi: 10.2807/1560-7917.ES.2018.23.1.17-00335

Carroll, L. M., Wiedmann, M., Mukherjee, M., Nicholas, D. C., Mingle, L. A., Dumas, N. B., et al. (2019). Characterization of emetic and diarrheal *Bacillus cereus* strains from a 2016 foodborne outbreak using whole-genome sequencing: Addressing the microbiological, epidemiological, and bioinformatic challenges. *Front Microbiol* 10, 144. doi: 10.3389/FMICB.2019.00144/FULL

Chan, Y. W., Hoban, A., Moore, H., Greig, D. R., Painset, A., Jorgensen, F., et al. (2023). Two Outbreaks of Foodborne Gastrointestinal Infection Linked to Consumption of Imported Melons, United Kingdom, March to August 2021. *J Food Prot* 86, 100027. doi: 10.1016/J.JFP.2022.100027

Chung, S. Y., Kwon, T., Bak, Y. S., Park, J. J., Kim, C. H., Cho, S. H., et al. (2019). Comparative genomic analysis of enterotoxigenic *Escherichia coli* O159 strains isolated from diarrheal patients in Korea. *Gut Pathog* 11, 9. doi: 10.1186/S13099-019-0289-6

Crowe, S. J., Green, A., Hernandez, K., Peralta, V., Bottichio, L., Defibaugh-Chavez, S., et al. (2017). Utility of Combining Whole Genome Sequencing with Traditional Investigational Methods To Solve Foodborne Outbreaks of *Salmonella* Infections Associated with Chicken: A New Tool for Tackling This Challenging Food Vehicle. *J Food Prot* 80, 654–660. doi: 10.4315/0362-028X.JFP-16-364

Di Febo, T., Schirone, M., Visciano, P., Portanti, O., Armillotta, G., Persiani, T., et al. (2019). Development of a Capture ELISA for Rapid Detection of *Salmonella enterica* in Food Samples. *Food Anal Methods* 12, 322–330. doi: 10.1007/S12161-018-1363-2/METRICS

Dong, Y., Wang, W., Jiang, T., Xu, J., Li, M., Yan, S., et al. (2022). A Family Outbreak of Type E Botulism Caused by Contaminated Vacuum-Packed Ambient-Stored Chili Chicken Feet in Zhangjiakou, China. *Foodborne Pathog Dis* 19, 613–621. doi: 10.1089/FPD.2022.0003

Ekman, C. C. J., Chiossi, M. F. do V., Meireles, L. R., de Andrade, H. F., Figueiredo, W. M., Marciano, M. A. M., et al. (2012). Case-control study of an outbreak of acute toxoplasmosis in an industrial plant in the state of São Paulo, Brazil. *Rev Inst Med Trop Sao Paulo* 54, 239–244. doi: 10.1590/S0036-46652012000500001

Elson, R., Awofisayo-Okuyelu, A., Greener, T., Swift, C., Painset, A., Amar, C. F. L., et al. (2019). Utility of Whole Genome Sequencing To Describe the Persistence and Evolution of *Listeria monocytogenes* Strains within Crabmeat Processing Environments Linked to Two Outbreaks of Listeriosis. *J Food Prot* 82, 30–38. doi: 10.4315/0362-028X.JFP-18-206

Espenhain, L., Riess, M., Müller, L., Colombe, S., Ethelberg, S., Litrup, E., et al. (2019). Cross-border outbreak of *Yersinia enterocolitica* O3 associated with imported fresh spinach, Sweden and Denmark, March 2019. *Eurosurveillance* 24, 1900368. doi: 10.2807/1560-7917.ES.2019.24.24.1900368

Fetsch, A., Contzen, M., Hartelt, K., Kleiser, A., Maassen, S., Rau, J., et al. (2014). *Staphylococcus aureus* food-poisoning outbreak associated with the consumption of ice-cream. *Int J Food Microbiol* 187, 1–6. doi: 10.1016/J.IJFOODMICRO.2014.06.017

Food Safety Authority, E., and Centre for Disease Prevention, E. (2024). The European Union One Health 2023 Zoonoses report. *EFSA Journal* 22, e9106. doi: 10.2903/J.EFSA.2024.9106

Gillesberg Lassen, S., Ethelberg, S., Björkman, J. T., Jensen, T., Sørensen, G., Kvistholm Jensen, A., et al. (2016). Two *Listeria* outbreaks caused by smoked fish consumption—using whole-genome sequencing for outbreak investigations. *Clinical Microbiology and Infection* 22, 620–624. doi: 10.1016/J.CMI.2016.04.017

Goodwin, S., McPherson, J. D., and McCombie, W. R. (2016). Coming of age: ten years of next-generation sequencing technologies. *Nature Reviews Genetics 2016 17:6* 17, 333–351. doi: 10.1038/nrg.2016.49

Gruber, J. F., Morris, S., Warren, K. A., Kline, K. E., Schroeder, B., Dettinger, L., et al. (2021). *Yersinia enterocolitica* Outbreak Associated with Pasteurized Milk. *Foodborne Pathog Dis* 18, 448–454. doi: 10.1089/FPD.2020.2924

Hardy, F. J., Johnson, A., Hamel, K., and Preece, E. (2015). Cyanotoxin bioaccumulation in freshwater fish, Washington State, USA. *Environ Monit Assess* 187. doi: 10.1007/S10661-015-4875-X

Hartantyo, S. H. P., Chau, M. L., Koh, T. H., Yap, M., Yi, T., Cao, D. Y. H., et al. (2020). Foodborne *Klebsiella pneumoniae*: Virulence Potential, Antibiotic Resistance, and Risks to Food Safety. *J Food Prot* 83, 1096–1103. doi: 10.4315/JFP-19-520

Heredia, N., and García, S. (2018). Animals as sources of food-borne pathogens: A review. *Animal Nutrition* 4, 250. doi: 10.1016/J.ANINU.2018.04.006

Inns, T., Ashton, P. M., Herrera-Leon, S., Lighthill, J., Foulkes, S., Jombart, T., et al. (2017). Prospective use of whole genome sequencing (WGS) detected a multi-country outbreak of *Salmonella* Enteritidis. *Epidemiol Infect* 145, 289–298. doi: 10.1017/S0950268816001941

Jajere, S. M. (2019). A review of *Salmonella enterica* with particular focus on the pathogenicity and virulence factors, host specificity and antimicrobial resistance including multidrug resistance. *Vet World* 12, 504. doi: 10.14202/VETWORLD.2019.504-521

Jenkins, C., Griffith, P., Hoban, A., Brown, C., Garner, J., Bardsley, M., et al. (2023). Foodborne Outbreak of Extended Spectrum Beta-lactamase Producing *Shigella sonnei* Associated with Contaminated Spring Onions in the United Kingdom. *J Food Prot* 86, 100074. doi: 10.1016/J.JFP.2023.100074

Jenkins, E., Cripe, J., Whitney, B. M., Greenlee, T., Schneider, B., Nguyen, T. A., et al. (2024). An Outbreak Investigation of *Salmonella* Weltevreden Illnesses in the United States Linked to Frozen Precooked Shrimp Imported from India – 2021. *J Food Prot* 87, 100360. doi: 10.1016/J.JFP.2024.100360

Jones, G., de la Gandara, M. P., Herrera-Leon, L., Herrera-Leon, S., Martinez, C. V., Hureaux-Roy, R., et al. (2019). Outbreak of *Salmonella enterica* serotype Poona in infants linked to persistent *Salmonella* contamination in an infant formula manufacturing facility, France, August 2018 to February 2019. *Eurosurveillance* 24, 1900161. doi: 10.2807/1560-7917.ES.2019.24.13.1900161

Kirk, M. D., Pires, S. M., Black, R. E., Caipo, M., Crump, J. A., Devleesschauwer, B., et al. (2015). World Health Organization Estimates of the Global and Regional Disease Burden of 22 Foodborne Bacterial, Protozoal, and Viral Diseases, 2010: A Data Synthesis. *PLoS Med* 12, e1001921. doi: 10.1371/JOURNAL.PMED.1001921

Labská, K., Špačková, M., Daniel, O., Včelák, J., Vlasáková, V., Černý, T., et al. (2021). A cross-border outbreak of *Salmonella* Bareilly cases confirmed by whole genome sequencing, Czech Republic and Slovakia, 2017 to 2018. *Eurosurveillance* 26, 2000131. doi: 10.2807/1560-7917.ES.2021.26.14.2000131

Lahti, E., Löfdahl, M., Ågren, J., Hansson, I., and Olsson Engvall, E. (2017). Confirmation of a Campylobacteriosis Outbreak Associated with Chicken Liver Pâté Using PFGE and WGS. *Zoonoses Public Health* 64, 14–20. doi: 10.1111/ZPH.12272

Lamichhane, B., Mawad, A. M. M., Saleh, M., Kelley, W. G., Harrington, P. J., Lovestad, C. W., et al. (2024). Salmonellosis: An Overview of Epidemiology, Pathogenesis, and Innovative Approaches to Mitigate the Antimicrobial Resistant Infections. *Antibiotics* 13, 76. doi: 10.3390/ANTIBIOTICS13010076

Lee, J., Lee, S., and Jiang, X. (2017). Cyanobacterial Toxins in Freshwater and Food: Important Sources of Exposure to Humans. *Annu Rev Food Sci Technol* 8, 281–304. doi: 10.1146/ANNUREV-FOOD-030216-030116

Lee, S. C., Blake Billmyre, R., Li, A., Carson, S., Sykes, S. M., Huh, E. Y., et al. (2014). Analysis of a foodborne fungal pathogen outbreak: Virulence and genome of a *Mucor circinelloides* isolate from yogurt. *mBio* 5. doi: 10.1128/mBio.01390-14

Li, X., Zhang, J., Zhang, H., Shi, X., Wang, J., Li, K., et al. (2022). Genomic analysis, antibiotic resistance, and virulence of *Staphylococcus aureus* from food and food outbreaks: A potential public concern. *Int J Food Microbiol* 377, 109825. doi: 10.1016/J.IJFOODMICRO.2022.109825

Lindsay, C., Flint, J., Lilly, K., Hope, K., Wang, Q., Howard, P., et al. (2018). Retrospective use of whole genome sequencing to better understand an outbreak of *Salmonella enterica* serovar Mbandaka in New South Wales, Australia. *Western Pac Surveill Response J* 9, 20. doi: 10.5365/WPSAR.2017.8.4.008

Logsdon, G. A., Vollger, M. R., and Eichler, E. E. (2020). Long-read human genome sequencing and its applications. *Nature Reviews Genetics 2020 21:10* 21, 597–614. doi: 10.1038/s41576-020-0236-x

Madad, A., Marshall, K. E., Blessington, T., Hardy, C., Salter, M., Basler, C., et al. (2023). Investigation of a Multistate Outbreak of *Listeria monocytogenes* Infections Linked to Frozen Vegetables Produced at Individually Quick-Frozen Vegetable Manufacturing Facilities. *J Food Prot* 86, 100117. doi: 10.1016/J.JFP.2023.100117

Majowicz, S. E., Scallan, E., Jones-Bitton, A., Sargeant, J. M., Stapleton, J., Angulo, F. J., et al. (2014). Global Incidence of Human Shiga Toxin–Producing *Escherichia coli* Infections and Deaths: A Systematic Review and Knowledge Synthesis. *Foodborne Pathog Dis* 11, 447. doi: 10.1089/FPD.2013.1704

McClure, M., Whitney, B., Gardenhire, I., Crosby, A., Wellman, A., Patel, K., et al. (2023). An Outbreak Investigation of *Salmonella* Typhimurium Illnesses in the United States Linked to Packaged Leafy Greens Produced at a Controlled Environment Agriculture Indoor Hydroponic Operation – 2021. *J Food Prot* 86, 100079. doi: 10.1016/J.JFP.2023.100079

Mitchell, M. R., Kirchner, M., Schneider, B., McClure, M., Neil, K. P., Madad, A., et al. (2024). Multistate outbreak of *Salmonella* Oranienburg infections linked to bulb onions imported from Mexico – United States, 2021. *Food Control* 160, 110325. doi: 10.1016/J.FOODCONT.2024.110325

Moffatt, C. R. M., Greig, A., Valcanis, M., Gao, W., Seemann, T., Howden, B. P., et al. (2016). A large outbreak of *Campylobacter jejuni* infection in a university college caused by chicken liver pâté, Australia, 2013. *Epidemiol Infect* 144, 2971. doi: 10.1017/S0950268816001187

Mörk, M. J., Karamehmedovic, N., Hansen, A., Öhd, J. N., Lindblad, M., Östlund, E., et al. (2022). Outbreak of *Salmonella* Newport linked to imported frozen cooked crayfish in dill brine, Sweden, July to November 2019. *Eurosurveillance* 27, 2100918. doi: 10.2807/1560-7917.ES.2022.27.22.2100918

Motladiile, T. W., Tumbo, J. M., Malumba, A., Adeoti, B., Masekwane, N. J., Mokate, O. M. R., et al. (2019). *Salmonella* food-poisoning outbreak linked to the National School Nutrition Programme, North West province, South Africa. *S Afr J Infect Dis* 34, 124. doi: 10.4102/SAJID.V34I1.124

Mottola, A., Bonerba, E., Bozzo, G., Marchetti, P., Celano, G. V., Colao, V., et al. (2016). Occurrence of emerging food-borne pathogenic *Arcobacter* spp. isolated from pre-cut (ready-to-eat) vegetables. *Int J Food Microbiol* 236, 33–37. doi: 10.1016/J.IJFOODMICRO.2016.07.012

Nazari Moghadam, M., Rahimi, E., Shakerian, A., and Momtaz, H. (2023). Prevalence of *Salmonella* Typhimurium and *Salmonella* Enteritidis isolated from poultry meat: virulence and antimicrobial-resistant genes. *BMC Microbiol* 23, 1–8. doi: 10.1186/S12866-023-02908-8/TABLES/6

Nichols, M., Conrad, A., Whitlock, L., Stroika, S., Strain, E., Weltman, A., et al. (2020). Short communication: Multistate outbreak of *Listeria monocytogenes* infections retrospectively linked to unpasteurized milk using whole-genome sequencing. *J Dairy Sci* 103, 176–178. doi: 10.3168/JDS.2019-16703

Nitnaware, K. M., Raskar, K. B., Agarwal, G., Chávez Montes, R. A., Chopra, R., López-Arredondo, D. L., et al. (2021). Whole-genome characterization and comparative genomics of a novel freshwater cyanobacteria species: *Pseudanabaena punensis*. *Mol Phylogenet Evol* 164, 107272. doi: 10.1016/J.YMPEV.2021.107272

Ohno, Y., Sekizuka, T., Kuroda, M., and Ikeda, T. (2023). Outbreaks of Campylobacteriosis Caused by Drinking Raw Milk in Japan: Evidence of Relationship Between Milk and Patients by Using Whole Genome Sequencing. *Foodborne Pathog Dis* 20, 375–380. doi: 10.1089/FPD.2023.0042

Paradis, A., Beaudet, M. F., Boisvert Moreau, M., and Huot, C. (2023). Investigation of a *Salmonella* Montevideo Outbreak Related to the Environmental Contamination of a Restaurant Kitchen Drainage System, Québec, Canada, 2020–2021. *J Food Prot* 86, 100131. doi: 10.1016/J.JFP.2023.100131

Pickova, D., Ostry, V., Toman, J., and Malir, F. (2021). Aflatoxins: History, significant milestones, recent data on their toxicity and ways to mitigation. *Toxins (Basel)* 13. doi: 10.3390/toxins13060399

Priyanka, B., Patil, R. K., and Dwarakanath, S. (2016). A review on detection methods used for foodborne pathogens. *Indian J Med Res* 144, 327–338. doi: 10.4103/0971-5916.198677

Ravindhiran, R., Sivarajan, K., Sekar, J. N., Murugesan, R., and Dhandapani, K. (2023). *Listeria monocytogenes* an Emerging Pathogen: a Comprehensive Overview on Listeriosis, Virulence Determinants, Detection, and Anti-Listerial Interventions. *Microbial Ecology 2023 86:4* 86, 2231–2251. doi: 10.1007/S00248-023-02269-9

Rhoads, A., and Au, K. F. (2015). PacBio Sequencing and Its Applications. *Genomics Proteomics Bioinformatics* 13, 278–289. doi: 10.1016/J.GPB.2015.08.002

Riley, L. W. (2020). Extraintestinal Foodborne Pathogens. *Annu Rev Food Sci Technol* 11, 275–294. doi: 10.1146/ANNUREV-FOOD-032519-051618

Rodriguez, C., Taminiau, B., Van Broeck, J., Delmée, M., and Daube, G. (2016). *Clostridium difficile* in Food and Animals: A Comprehensive Review. *Adv Exp Med Biol* 932, 65–92. doi: 10.1007/5584_2016_27

Rosen, H. E., Kimura, A. C., Crandall, J., Poe, A., Nash, J., Boetzer, J., et al. (2020). Foodborne Botulism Outbreak Associated With Commercial Nacho Cheese Sauce From a Gas Station Market. *Clinical Infectious Diseases* 70, 1695–1700. doi: 10.1093/CID/CIZ479

Ruiz de Alegría Puig, C., Fernández Martínez, M., Pablo Marcos, D., Agüero Balbín, J., and Calvo Montes, J. (2023). Outbreak of *Arcobacter butzleri*? An emerging enteropathogen. *Enfermedades infecciosas y microbiologia clinica (English ed.)* 41, 169–172. doi: 10.1016/J.EIMCE.2021.10.012

Saleh, I., Zeidan, R., and Abu-Dieyeh, M. (2024). The characteristics, occurrence, and toxicological effects of alternariol: a mycotoxin. *Arch Toxicol* 98, 1659–1683. doi: 10.1007/s00204-024-03743-0

Saravanan, A., Kumar, P. S., Hemavathy, R. V., Jeevanantham, S., Kamalesh, R., Sneha, S., et al. (2020). Methods of detection of foodborne pathogens: a review. *Environmental Chemistry Letters 2020 19:1* 19, 189–207. doi: 10.1007/S10311-020-01072-Z

Seelman Federman, S., Jenkins, E., Wilson, C., DeLaGarza, A., Schwensohn, C., Schneider, B., et al. (2024). An investigation of an outbreak of *Salmonella* Typhimurium infections linked to cantaloupe – United States, 2022. *Food Control* 166, 110733. doi: 10.1016/J.FOODCONT.2024.110733

Siddique, A., Tauqeer, A., Ali, A., Ahsan, A., Iqbal, S., Patel, A., et al. (2024). Whole-genome sequencing of three ciprofloxacin-resistant *Salmonella* Reading (ST93) strains, an emerging *Salmonella* serovar in the poultry sector of Pakistan. *Microbiol Resour Announc* 13. doi: 10.1128/MRA.00006-24

Silva, A. J., Yang, Z., Wolfe, J., Hirneisen, K. A., Ruelle, S. B., Torres, A., et al. (2021). Application of whole-genome sequencing for *norovirus* outbreak tracking and surveillance efforts in Orange County, CA. *Food Microbiol* 98, 103796. doi: 10.1016/J.FM.2021.103796

Simon, S., Trost, E., Bender, J., Fuchs, S., Malorny, B., Rabsch, W., et al. (2018). Evaluation of WGS based approaches for investigating a foodborne outbreak caused by *Salmonella enterica* serovar Derby in Germany. *Food Microbiol* 71, 46–54. doi: 10.1016/J.FM.2017.08.017

Smith, A. M., Tau, N. P., Ngomane, H. M., Sekwadi, P., Ramalwa, N., Moodley, K., et al. (2020). Whole-genome sequencing to investigate two concurrent outbreaks of *Salmonella* enteritidis in South Africa, 2018. *J Med Microbiol* 69, 1303–1307. doi: 10.1099/JMM.0.001255/CITE/REFWORKS

Socioeconomic – Harmful Algal Blooms (n.d.). Available at: https://hab.whoi.edu/impacts/impacts-socioeconomic/ (Accessed December 6, 2024).

Souii, A., M’hadheb-Gharbi, M. Ben, and Gharbi, J. (2016). Nucleic acid-based biotechnologies for foodborne pathogen detection using routine time-intensive culture-based methods and fast molecular diagnostics. *Food Sci Biotechnol* 25, 11–20. doi: 10.1007/S10068-016-0002-1/METRICS

Sundararaman, B., Shapiro, K., Packham, A., Camp, L. E., Meyer, R. S., Shapiro, B., et al. (2024). Whole genome enrichment approach for genomic surveillance of *Toxoplasma gondii*. *Food Microbiol* 118, 104403. doi: 10.1016/J.FM.2023.104403

Tong, W., Yang, D., Qiu, S., Tian, S., Ye, Z., Yang, S., et al. (2024). Relevance of genetic causes and environmental adaptation of *Cronobacter* spp. isolated from infant and follow-up formula production factories and retailed products in China: A 7-year period of continuous surveillance based on genome-wide analysis. *Science of The Total Environment* 946, 174368. doi: 10.1016/J.SCITOTENV.2024.174368

Uljanovas, D., Gölz, G., Fleischmann, S., Kudirkiene, E., Kasetiene, N., Grineviciene, A., et al. (2023). Genomic Characterization of *Arcobacter butzleri* Strains Isolated from Various Sources in Lithuania. *Microorganisms* 11, 1425. doi: 10.3390/MICROORGANISMS11061425/S1

Umeda, K., Ono, H. K., Wada, T., Motooka, D., Nakamura, S., Nakamura, H., et al. (2021). High production of egc2-related staphylococcal enterotoxins caused a food poisoning outbreak. *Int J Food Microbiol* 357, 109366. doi: 10.1016/J.IJFOODMICRO.2021.109366

van Dijk, E. L., Jaszczyszyn, Y., Naquin, D., and Thermes, C. (2018). The Third Revolution in Sequencing Technology. *Trends Genet* 34, 666–681. doi: 10.1016/J.TIG.2018.05.008

Wang, Q., Han, Y. Y., Zhang, T. J., Chen, X., Lin, H., Wang, H. N., et al. (2024). Whole-genome sequencing of *Escherichia coli* from retail meat in China reveals the dissemination of clinically important antimicrobial resistance genes. *Int J Food Microbiol* 415, 110634. doi: 10.1016/J.IJFOODMICRO.2024.110634

Wang, Y., Zhao, Y., Bollas, A., Wang, Y., and Au, K. F. (2021). Nanopore sequencing technology, bioinformatics and applications. *Nature Biotechnology 2021 39:11* 39, 1348–1365. doi: 10.1038/s41587-021-01108-x

Wenger, A. M., Peluso, P., Rowell, W. J., Chang, P. C., Hall, R. J., Concepcion, G. T., et al. (2019). Accurate circular consensus long-read sequencing improves variant detection and assembly of a human genome. *Nat Biotechnol* 37, 1155–1162. doi: 10.1038/S41587-019-0217-9

Yang, S. C., Lin, C. H., Aljuffali, I. A., and Fang, J. Y. (2017). Current pathogenic *Escherichia coli* foodborne outbreak cases and therapy development. *Archives of Microbiology 2017 199:6* 199, 811–825. doi: 10.1007/S00203-017-1393-Y

Yao, L., Cooper, A., Lau, C. H. F., Wong, A., Blais, B. W., and Carrillo, C. D. (2024). Strain-specific Recovery of *S. sonnei* from Artificially Contaminated Baby Carrots: Enhancing Food-safety Investigations with a Customized *Shigella* Detection Method Based on Genomically predicted Antibiotic Resistance Traits. *J Food Prot* 87, 100300. doi: 10.1016/J.JFP.2024.100300

Ye, Z., Wang, L., Liu, T., Li, F., Liu, Y., Li, Z., et al. (2024). The prevalence, antimicrobial resistance and molecular genetic characteristics of fosfomycin-resistant *Klebsiella pneumoniae* isolated from raw milk. *Int Dairy J* 159, 106068. doi: 10.1016/J.IDAIRYJ.2024.106068

Yong, W., Guo, B., Shi, X., Cheng, T., Chen, M., Jiang, X., et al. (2018). An investigation of an acute gastroenteritis outbreak: *Cronobacter sakazakii*, a potential cause of food-borne illness. *Front Microbiol* 9, 2549. doi: 10.3389/FMICB.2018.02549/FULL

Zhang, Y., Chen, L., Jiang, Y., Yang, B., Chen, J., Zhan, L., et al. (2022). Epidemiological and Whole-Genome Sequencing Analysis of a Gastroenteritis Outbreak Caused by a New Emerging Serotype of *Vibrio parahaemolyticus* in China. *Foodborne Pathog Dis* 19, 550–557. doi: 10.1089/FPD.2022.0002

Zhong, X., Pan, Z., Mu, Y., Zhu, Y., Zhang, Y., Ma, J., et al. (2022). Characterization and epidemiological analysis of *Vibrio parahaemolyticus* isolated from different marine products in East China. *Int J Food Microbiol* 380, 109867. doi: 10.1016/J.IJFOODMICRO.2022.109867
